# Efficacy of indocyanine green fluorescence imaging-guided lymphadenectomy in radical gastrectomy for gastric cancer: A systematic review and meta-analysis

**DOI:** 10.3389/fonc.2022.998159

**Published:** 2022-10-18

**Authors:** Bo Dong, Anyuan Zhang, Yuqiang Zhang, Wei Ye, Lan Liao, Zonglin Li

**Affiliations:** ^1^ Department of General Surgery, The People’s Hospital of Rongchang District, Chongqing, China; ^2^ Department of Gastrointestinal Surgery, The Affiliated Hospital of Southwest Medical University, Luzhou, China

**Keywords:** gastric cancer, lymphadenectomy, indocyanine green, fluorescence imaging, minimally invasive surgery

## Abstract

**Background:**

Indocyanine green (ICG) imaging-guided lymphadenectomy has been introduced in gastric cancer (GC) surgery and its clinical value remains controversial. The aim of this study is to evaluate the efficacy of ICG fluorescence imaging-guided lymphadenectomy in radical gastrectomy for GC.

**Methods:**

Studies comparing lymphadenectomy in radical gastrectomy between use and non-use of ICG fluorescence imaging up to July 2022 were systematically searched from PubMed, Web of Science, Embase and Cochrane Library. A pooled analysis was performed for the available data regarding the baseline features, the number of retrieved lymph nodes (LNs), the number of metastatic LNs and surgical outcomes as well as oncological outcomes. RevMan 5.3 software was used to perform the statistical analysis. Quality evaluation and publication bias were also conducted.

**Results:**

17 studies with a total of 2274 patients (1186 in the ICG group and 1088 in the control group) undergoing radical gastrectomy and lymphadenectomy were included. In the pooled analysis, the baseline features were basically comparable. However, the number of retrieved LNs in the ICG group was significantly more than that in the control group (MD = 7.41, 95% CI = 5.44 to 9.37, *P* < 0.00001). No significant difference was found between the ICG and control groups in terms of metastatic LNs (MD = -0.05, 95% CI = -0.25 to 0.16, *P* = 0.65). In addition, the use of ICG could reduce intraoperative blood loss (MD = -17.96, 95% CI = -27.89 to -8.04, *P* = 0.0004) without increasing operative time (*P* = 0.14) and overall complications (*P* = 0.10). In terms of oncological outcomes, the use of ICG could reduce the overall recurrence rate (OR = 0.50; 95% CI 0.28-0.89; *P* = 0.02) but could not increase the 2-year overall survival rate (OR = 1.25; 95% CI 0.72-2.18; *P* = 0.43).

**Conclusions:**

ICG imaging-guided lymphadenectomy is valuable for complete LNs dissection in radical gastrectomy for GC. However, more high-quality randomized controlled trials are needed to confirm this benefit.

## Introduction

Gastric cancer (GC) is one of the most common cancers worldwide with more than one million new cases and 760,000 deaths in 2020 (1). At present, radical gastrectomy combined with D2 lymphadenectomy is still the most effective treatment for GC (2, 3). The status of lymph nodes (LNs) is a stronger prognostic factor for the survival of GC patients and sufficient lymphadenectomy can improve the prognosis of GC patients (4–6). Howerer, lymphadenectomy for GC is usually performed without the aid of visual instruments and complete lymphadenectomy is sometimes difficult, especially for inexperienced gastrointestinal surgeons, which always results in LNs residue and in turn leads to tumor recurrence as well as the death of these patient. Therefore, the application of intraoperative navigation technology to assist systematic and complete lymphadenectomy is essential for radical gastrectomy.

Indocyanine green (ICG), a lymphatic tracer with minimal adverse effects, can bind intensely with serum proteins *in vivo* and emits fluorescence on exposure to near-infrared rays of wavelength 760-780 nm (7, 8). In recent years, ICG fluorescence imaging for LNs tracing has attracted surgeons’ attention and ICG imaging-guided lymphadenectomy has been introduced in GC surgery (9–11). Until now, several studies have reported that ICG imaging-guided lymphadenectomy was applied to GC surgery and showed promising results in increasing the number of retrieved LNs, without increasing operative time and overall complications (12–14). However, whether ICG imaging-guided lymphadenectomy is indeed beneficial for LNs dissection remains unclear. Therefore, further research is needed to validate the efficacy of ICG imaging-guided lymphadenectomy in radical gastrectomy for GC.

The aim of this meta-analysis is to evaluate the efficacy of ICG imaging-guided lymphadenectomy in radical gastrectomy for GC based on the current published studies.

## Methods

This meta-analysis was carried out in line with the Preferred Reporting Items for Systematic Reviews and Meta-Analysis (PRISMA) statement.

### Search strategy

Studies comparing lymphadenectomy in radical gastrectomy between use and non-use of ICG fluorescence imaging up to July 2022 were systematically searched from PubMed, Web of Science, Embase and Cochrane Library. The keywords used for the search were “gastric cancer”, “lymphadenectomy” and “ICG”. Thus, the following search string was used across the above databases: [“gastric cancer” OR “gastric carcinoma” OR “gastric tumor” OR “stomach cancer” OR “stomach carcinoma” OR “stomach tumor”] AND [“lymphadenectomy” OR “lymph node excision” OR “lymph node dissection”] AND [“indocyanine green” OR “ICG”]. Articles from previously published reviews were also checked for potential articles. The search was conducted independently by two authors (BD and AZ). The search was last performed on July 3, 2022.

### Study selection and data extraction

The included studies met the following criteria: (1) GC patients with laparoscopic or robotic surgery; (2) lymphadenectomy performed in accordance with the guidelines for the treatment of GC; (3) comparative studies about lymphadenectomy in radical gastrectomy between use and non-use of ICG fluorescence imaging; (4) studies with reported outcome including the number of retrieved LNs in the ICG and control groups; (5) original research published in English. The exclusion criteria were as follows: (1) studies published as reviews, comments, letters, case reports, animal studies and meeting abstracts; (2) studies without the outcome about the number of retrieved LNs; (3) unavailability of effective data for meta-analysis.

Two reviewers (BD and AZ) carried out the screening and extraction process independently. First, studies were screened by titles and abstract. Then, the potential studies were checked for full text. For the eligible articles, the following information from each article was recorded: first author, publication year, country, study interval, study design, study object, sample size, extent of lymphadenectomy, ICG dosage and imaging system. Furthermore, the following clinicopathological parameters were extracted from these studies: sex, age, body mass index (BMI), American Society of Anesthesiologists (ASA) score, tumor size, pathological stage, histologic type, method of gastrectomy, neoadjuvant chemoradiotherapy, the number of retrieved LNs, the number of metastatic LNs, operation time, intraoperative blood loss, overall complications, overall recurrence rate and 2-year overall survival (OS) rate. Results were checked by a third author (ZL).

### Risk of bias assessment

Qualities of the selected studies were assessed according to the Cochrane Handbook. Biases including selection, performance, detection, attrition, reporting and others were evaluated and the outcomes were summarized in the form of a bias graph.

### Statistical analysis

The odds ratio (OR) and mean difference (MD) with their 95% confidence interval (CI) were used as the effect size for dichotomous and continuous variables, respectively. For studies that only reported median and range, data were converted into mean and standard deviation (SD) following the method reported by Hozo SP et al. (15). Heterogeneity among studies was assessed by χ^2^ and I^2^ statistics. fixed-effects models and random-effects models were used in cases of nonsignificant (I^2^ ≤ 50%) and significant (I^2^ > 50%) heterogeneity, respectively. For the assessment of publication bias, a funnel plot was conducted. A *P* value < 0.05 was considered significant. All of the statistical analyses were performed by RevMan 5.3 software (Cochrane, London, UK).

## Results

### Characteristics of studies

**Figure 1:**
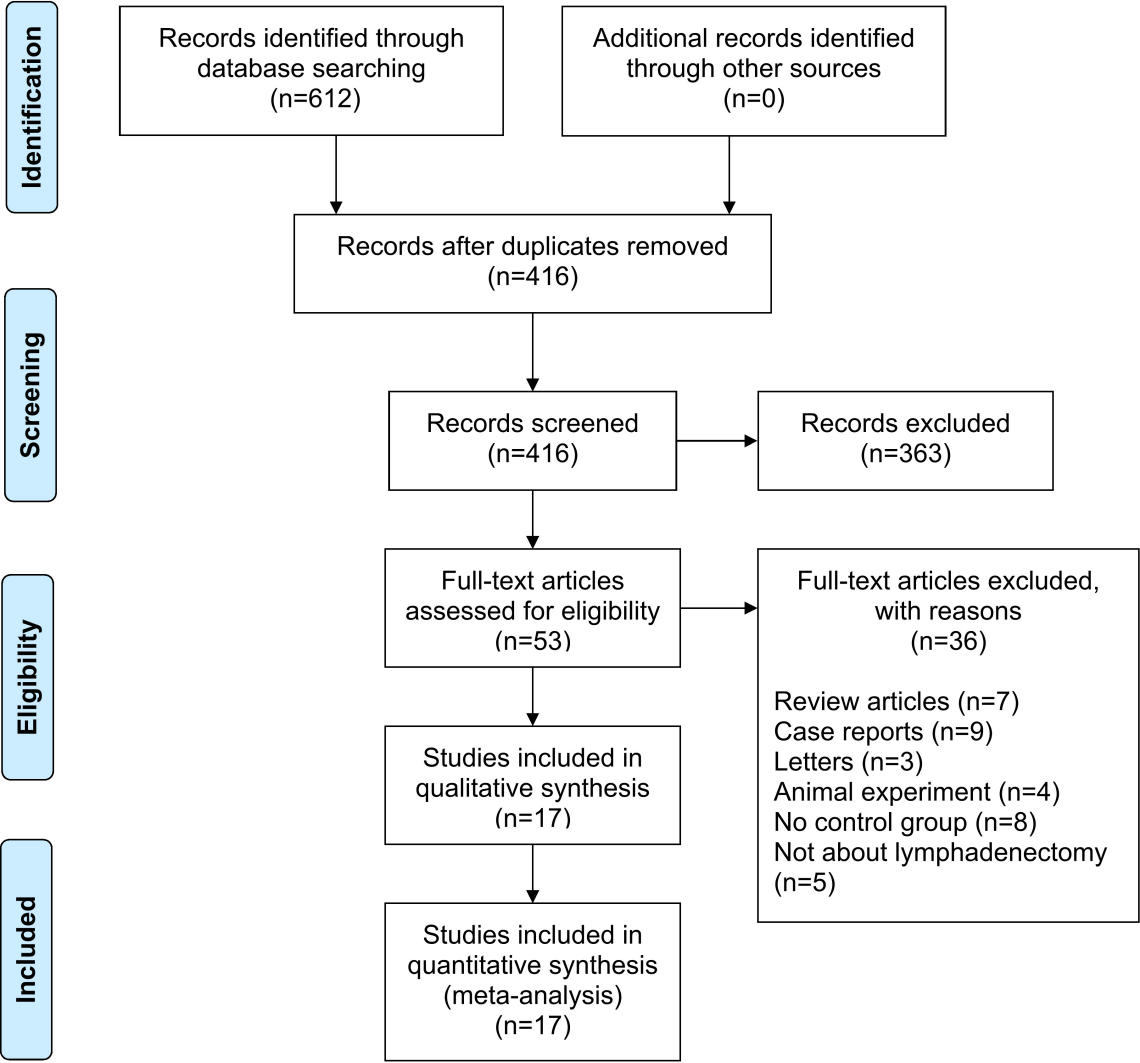
PRISMA flowchart of literature search and selection process. *PRISMA* preferred reporting items for systematic review and meta-analysis.

**Figure 2:**
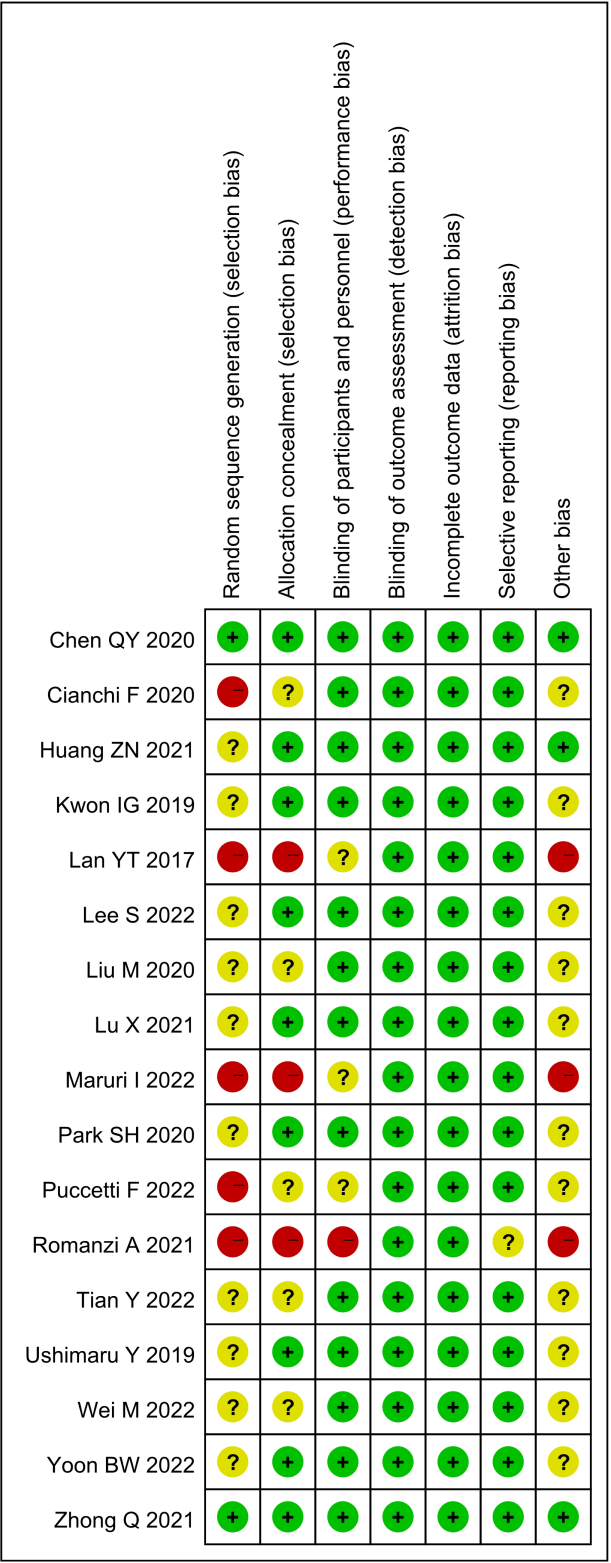
Risk of bias summary for the included studies.

A total of 612 studies were identified, and 17 studies including 15 retrospective studies and 2 randomized controlled trials (RCTs) were ultimately included in this meta-analysis (13, 14, 16–30). The details of the selection procedures are shown to be in line with the PRISMA flowchart (**Figure 1**). General information from those included studies is summarized in **Table 1**. The total number of GC patients included was 2274 (1186 in the ICG group and 1088 in the control group). These studies were from five countries (i.e., China, Italy, Korea, Spain and Japan) and were published from 2017 to 2022. The sample size ranged from 20 to 514 patients. Laparoscopic or robotic radical total or distal gastrectomy combined with D1+ or D2 lymphectomy were performed in these studies. Nevertheless, the dosage of ICG and imaging systems considered differed in these studies. According to the Cochrane Handbook, the 17 studies were at slight or moderate risk of bias. The items evaluated for each study are shown in **Figure 2**.

**Table 1:** Characteristics of studies.

Reference	Country	Study interval	Study object	Study design	Sample size (ICG: Control)	Method of gastrectomy	Extent of lymphadenectomy	ICG dosage	ICG injection method	ICG injection time	ICG imaging system	Outcomes
Chen QY (13)	China	2018-2019	pT1-4aN0-3M0	S;RCT	129: 129	laparoscopic TG and DG	D2	2.5 mg	endoscopic submucosal injection	1 day before surgery	Stryker	1, 2, 3, 4, 5
Cianchi F (14)	Italy	2014-2018	pT1-3N0-3M0	S;R	37: 37	laparoscopic TG and DG	D2	2.5 mg	endoscopic submucosal injection	1 day before surgery	Firefly	1, 2, 3, 5
Huang ZN (16)	China	2010-2020	cT1-4N0-3M0	M;R;PSM	94: 94	laparoscopic TG and DG	D2	4.5 mg	subserosal injection	intraoperative	Stryker	1, 4, 5
Kwon IG (17)	Korea	2012-2014	pT1-2N0-1M0	S;R;PSM	40: 40	robotic TG and DG	D1+ or D2	3 mg	endoscopic submucosal injection	1 day before surgery	NA	1, 3, 4, 5
Lan YT (18)	China	2011-2016	pT1-4N0-3M0	S;R	14: 65	robotic TG and DG	D1+ or D2	6 mg	subserosal injection	intraoperative	NA	1, 3, 4, 5
Lee S (19)	Korea	2013-2018	pT1-4aN0-3M0	S;R	74: 94	laparoscopic or robotic TG	D2 + No. 10	1.5-3.0 mg	endoscopic submucosal injection	1 day before surgery	Firefly and Pinpoint	1, 3, 4, 5, 6, 7
Liu M (20)	China	2017-2019	pT1-4N0-3M0	S;R	61: 75	laparoscopic DG	D2	1.25 mg	endoscopic submucosal injection	20 to 30 hours before surgery	Stryker	1, 2, 3, 4, 5
Lu X (21)	China	2015-2019	pT1-4N0-3M0	S;R;PSM	28: 28	laparoscopic TG, DG and PG	D2	2.5 mg	endoscopic submucosal injection	intraoperative	Pinpoint	1, 3, 4, 5, 6
Maruri I (22)	Spain	2018-2019	cT1-4N0-3M0	S;R	17: 17	laparoscopic TG and DG	D1+ or D2	3 mg	endoscopic submucosal injection	18 to 24 hours before surgery	NA	1, 2, 6
Park SH (23)	Korea	2017-2018	pT1-4N0-3M0	S;R;PSM	20: 60	laparoscopic DG	D1+ or D2	0.5 mg	endoscopic submucosal injection	intraoperative	Pinpoint	1, 3, 4, 5
Puccetti F (24)	Italy	2015-2021	pT1-3N0-3M0	S;R	38: 64	laparoscopic TG	D2	0.25 mg	endoscopic submucosal injection	12 to 24 hours before surgery	NA	1, 2, 3
Romanzi A (25)	Italy	2018-2019	pT1-4bN0-3M0	S;R	10: 10	robotic DG	D2	3 mg	endoscopic submucosal injection	18 hours before surgery	Firefly	1, 3
Tian Y (26)	China	2019-2020	NA	S;R	27: 32	robotic DG	D2	5 mg	endoscopic submucosal injection	1 day before surgery	NA	1, 3, 4, 5
Ushimaru Y (27)	Japan	2015-2017	pT1-4N0-3M0	S;R;PSM	84: 84	laparoscopic TG and DG	D1+ or D2	0.1 mg	endoscopic submucosal injection	1 day before surgery	STORZ	1, 3, 4, 5
Wei M (28)	China	2018-2019	pT1-4aN0-3M0	S;R	107: 88	laparoscopic TG and DG	D2	2.5 mg	endoscopic submucosal injection	12 to 24 hours before surgery	Stryker	1, 2, 3, 4, 5, 6, 7
Yoon BW (29)	Korea	2010-2020	pT1-4aN0-3M0	S;R;PSM	21: 42	laparoscopic DG	D2	0.4 mg	endoscopic submucosal injection	1 day before surgery	NA	1, 2, 3
Zhong Q (30)	China	2018-2020	pT1-4aN0-3M0	M;RCT	385: 129	laparoscopic TG and DG	D2	4.5 mg	subserosal injection	intraoperative	Stryker	1, 2

ICG, indocyanine green; S single centre; M, multicentre; R, retrospective study; PSM, propensity score matching; RCT, randomized controlled trial; NA, not available, 1= number of retrieved lymph nodes, 2= number of metastatic lymph nodes, 3=operative time, 4=intraoperative blood loss, 5=overall complications, 6=overall recurrence rate, 7 = 2-year overall survival.

### Patient- and tumor-related baseline characteristics

**Figure 3:**
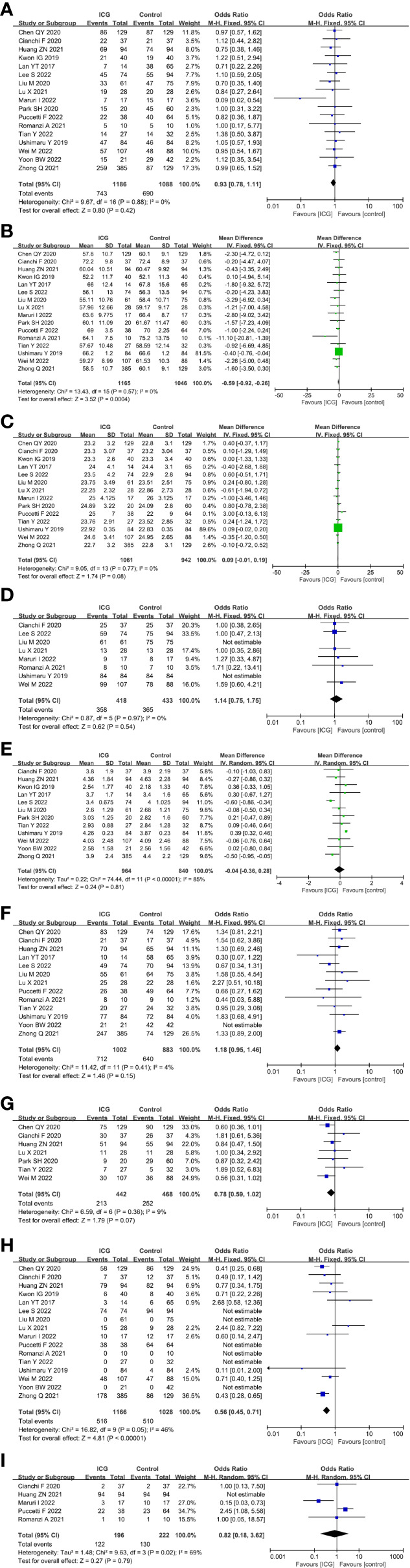
Forest plots showing the assessment of baseline features including **(A)** sex, **(B)** age, **(C)** body mass index, **(D)** American Society of Anaesthesiologists score, **(E)** tumor size, **(F)** pathological stage, **(G)** histologic type, **(H)** method of gastrectomy, **(I)** neoadjuvant chemoradiotherapy. *ICG*, indocyanine green.

For the patient- and tumor-related variables, sex (male and female), age (mean ± SD), BMI (mean ± SD), ASA score (ASA 1/2 and ASA 3/4), tumor size (mean ± SD), pathological stage (stage 1/2 and stage 3/4), histologic type (differentiated and other types), method of gastrectomy (total gastrectomy and distal gastrectomy) and neoadjuvant chemoradiotherapy (with and without) were analyzed. Except for age (*P* = 0.0004) and the method of gastrectomy (*P* < 0.00001), other variables were all comparable between the ICG and control groups (*P* > 0.05) analysed by the fixed-effects models (I^2^ ≤ 50%) and random-effects models (I^2^ > 50%). The baseline parameters between the two groups were basically statistically insignificant, as shown in **Figure 3**.

### Efficacy of lymphadenectomy

**Figure 4:**
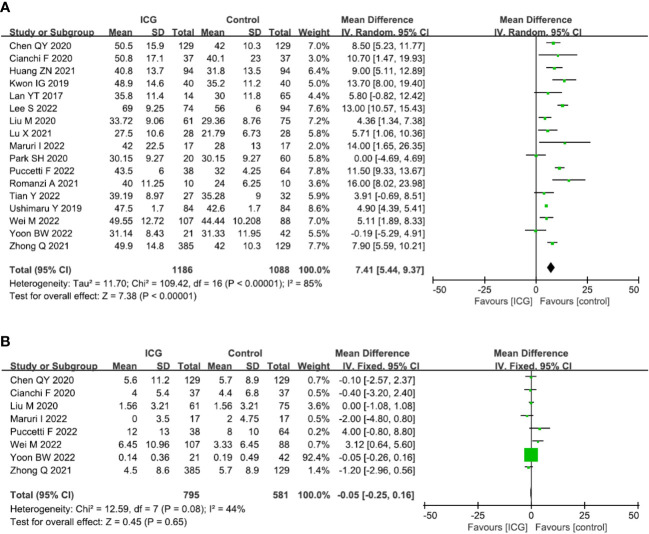
Forest plots showing the assessment of lymphadenectomy including **(A)** the number of retrieved lymph nodes, **(B)** the number of metastatic lymph nodes. ***ICG*,** indocyanine green.

The primary outcome of this study was to assess the efficacy of lymphadenectomy by using ICG fluorescence imaging. Ultimately, 17 studies (2274 patients) (13, 14, 16–30) reporting this outcome were included in our meta-analysis. The pooled analysis revealed that the number of retrieved LNs in the ICG group was significantly more than that in the control group (MD = 7.41, 95% CI = 5.44 to 9.37, *P* < 0.00001) (**Figure 4A**), but there is no significant difference in terms of metastatic LNs between the ICG and control groups (MD = -0.05, 95% CI = -0.25 to 0.16, *P* = 0.65) (**Figure 4B**).

### Surgical outcomes

**Figure 5:**
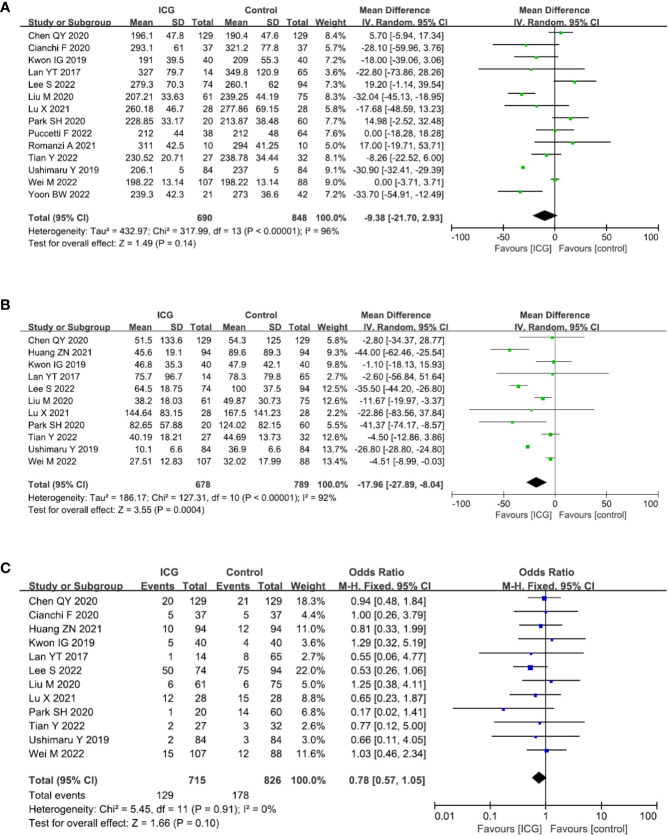
Forest plots showing the assessment of surgical outcomes including **(A)** operative time, **(B)** intraoperative blood loss, **(C)** overall complication. *ICG*, indocyanine green.

14 studies (13, 14, 17–21, 23–29) reported the operation time and the pooled analysis showed no difference between the ICG and control groups (MD = −9.38, 95% CI = −21.70 to 2.93, *P* = 0.14) (**Figure 5A**). However, 11 studies (13, 16–21, 23, 26–28) reported the intraoperative blood loss and showed that the use of ICG could reduce intraoperative blood loss (MD = -17.96, 95% CI = -27.89 to -8.04, *P* = 0.0004) (**Figure 5B**). 12 studies (13, 14, 16–21, 23, 26–28) reported the overall complications and there was a trend that the use of ICG was related to less overall complications with no statistic difference (OR = 0.78, 95% CI = 0.57 to 1.05, *P* = 0.10) (**Figure 5C**).

### Oncological outcomes

**Figure 6:**
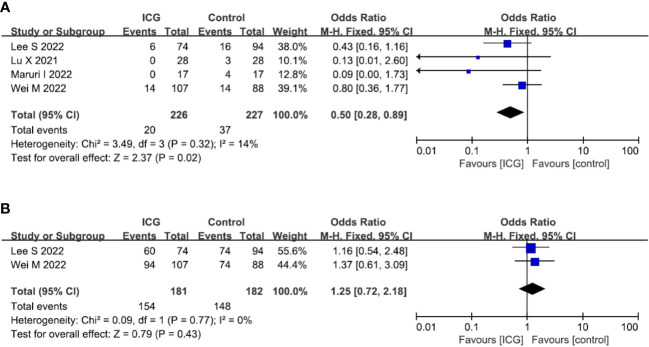
Forest plots showing the assessment of oncological outcomes including **(A)** overall recurrence rate, **(B)** 2-year OS rate. *ICG*, indocyanine green; *OS*, overall survival.

In terms of oncological outcomes, four studies (19, 21, 22, 28) reported the overall recurrence rate and the pooled analysis showed that the use of ICG could reduce the overall recurrence rate (OR = 0.50; 95% CI 0.28-0.89; *P* = 0.02) (**Figure 6A**). However, in terms of postoperative overall survival, two studies (19, 28) reported the 2-year overall survival rate but there was no difference between the ICG and control groups (OR = 1.25; 95% CI 0.72-2.18; *P* = 0.43) (**Figure 6B**).

### Publication bias

**Figure 7:**
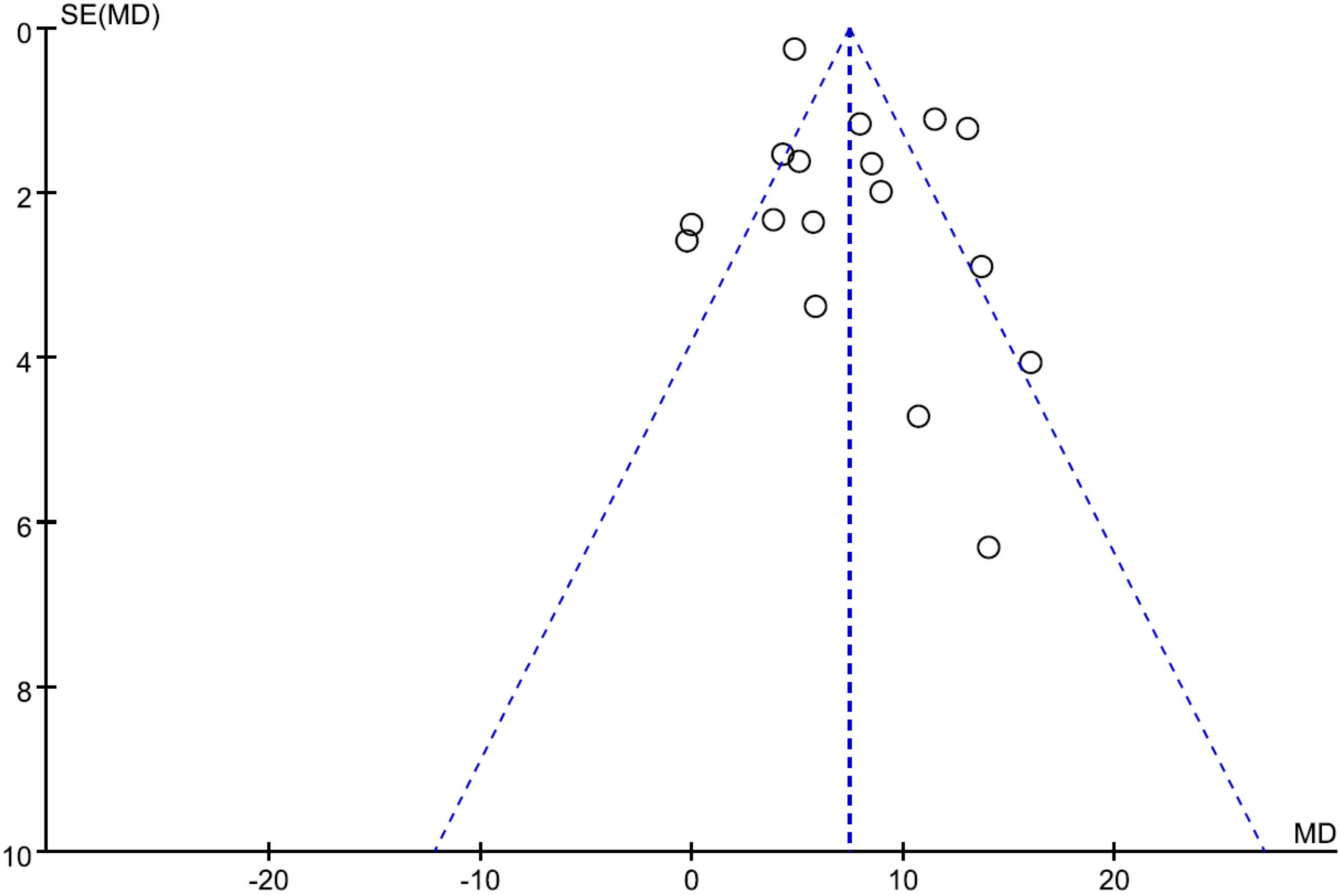
Funnel plots of publication bias for the number of retrieved lymph nodes.

The funnel plot was used to assess potential publication bias in the meta-analysis of the correlation between the use of ICG fluorescence imaging and the number of retrieved LNs. As shown in **Figure 7**, the funnel plot was symmetrical, which showed a low risk of publication bias in this study.

## Discussion

GC is one of the most common malignant tumors of digestive tract and radical surgery is the mainstay of treatment, which involves performing gastric resection with negative margins and adequate systemic LNs dissection. The status of LNs is a stronger prognostic factor for the survival of GC patients and radical lymphadenectomy can significantly improve the long-term survival (31, 32). In addition, whether or not the resected LNs have metastasis, complete perigastric lymphadenectomy is important for the accurate staging of tumors and the decision of subsequent treatment (33–35). So the retrieval of more LNs in radical gastrectomy has become the special requirement for gastrointestinal surgeons.

Currently, minimally invasive surgery, including laparoscopic and robotic methods, has been widely used in the treatment of GC, especially for early GC (36, 37). However, the oncological efficacy of minimally invasive techniques for the treatment of advanced GC is still controversial because of the concern about not being able to perform an accurate D2 lymphadenectomy and the oncological safety (38, 39). At present, lymphadenectomy in radical gastrectomy is often performed depending on the surgeon’s experience and without the aid of visual instruments. However, due to the complex lymphatic drainage and abundant LNs around the stomach, it is often difficult for surgeons, especially for those younger and inexperienced surgeons, to perform an accurate and effective D2 lymphadenectomy without increasing surgical complications.

In recent years, ICG fluorescence imaging for LNs tracing has attracted surgeons’ attention and ICG imaging-guided lymphadenectomy has been introduced in GC surgery. Chen QY et al. (13) performed a RCT and indicated that ICG can noticeably improve the number of retrieved LNs without increased complications in GC patients undergoing D2 lymphadenectomy and they recommend ICG fluorescence imaging should be performed for routine lymphatic mapping during laparoscopic gastrectomy, especially total gastrectomy. Kwon et al. (17) also reported that ICG-guided lymphadenectomy is effective in retrieving more LNs than conventional surgery and had a similar incidence of postoperative complications to conventional surgery. Lee S et al. (19) point out ICG fluorescence imaging-guided lymphadenectomy is an effective tool for complete LNs dissection at the splenic hilum and it may help select patients who do not need splenic hilar LNs dissection during a total gastrectomy. However, Lan et al. (18) reported that the number of retrieved LNs in the ICG group was not improved compared with the non-ICG group. According to the pooled analysis in our study, the number of retrieved LNs in the ICG group was significantly more than that in the control group (*P* < 0.00001) and the use of ICG could reduce intraoperative blood loss (*P* = 0.0004) without increasing operative time (*P* = 0.14) and overall complications (*P* = 0.10). Theoretically, total gastrectomy could obtain more LNs than distal gastrectomy. In our combined analysis, the proportion of total gastrectomy in the ICG group is lower than that in the control group (44.3% vs. 49.6%), but more LNs were obtained, which further indicated that ICG fluorescence imaging-guided lymphadenectomy could increased the number of retrieved LNs. Also, Yoon BW et al. (29) reported that the use of ICG could secure the oncologically safe of proximal resection margin in totally laparoscopic distal gastrectomy, with the advantage of reducing the operation time and has the benefit of locating the tumor. These results suggest that the ICG fluorescence imaging-guided lymphadenectomy is valuable in terms of LNs dissection and short-term outcomes. Nevertheless, the present meta-analysis demonstrated that there was no significant difference in metastatic LNs between the ICG and control groups. The reasons for this outcome may be explained as follows: (1) The metastatic LNs can be removed completely by standard D2 lymphectomy without the use of ICG imaging-guided lymphadenectomy, and (2) Some researchers removed all the fluorescent LNs, even these LNs were outside the extent of D2 lymphectomy (13).

Reducing postoperative tumor recurrence and prolonging patients’ survival time are the ultimate goals of standardized and systematic lymphectomy (40–42). Lees et al. (19) reported that ICG fluorescence imaging-guided lymphadenectomy could reduce the tumor recurrence rate after surgery, with the recurrence rate 8.1% and 17.0% in the ICG and control groups, respectively. And another two studies also got the similar results (21, 22). However, Wei M et al. (28) pointed out that the tumor recurrence rates were similar between the two groups after surgery, with the recurrence rate 13.1% and 15.9% in the ICG and control groups, respectively. According to the pooled analysis, ICG fluorescence imaging-guided lymphadenectomy could reduce the overall recurrence rate (*P* = 0.02). However, the 2-year OS rates were comparable between the ICG and control groups (*P* = 0.43). Nevertheless, this result does not indicate that ICG fluorescence imaging-guided lymphadenectomy cannot improve the prognosis of GC patients, because there were only two studies reported survival results, and the follow-up period was shorter, without 5-year survival rate. So more studies with longer follow-up are necessary and expected.

Our study has some limitations. Firstly, there were only two RCTs in the included studies, which may increase the risk of selective bias. Therefore, more high-quality RCTs are expected to provide more credible evidence on this issue. Secondly, due to the limitations of data acquisition and language understanding, only English studies were included in this meta-analysis, which may also increase the risk of selective bias. Thirdly, the uses of ICG, including the dosage, injection method, injection time and ICG imaging system, were all different in these studies, which probably led to heterogeneity in the outcomes.

## Conclusions

Despite the limitations of the included studies, this meta-analysis indicates that ICG fluorescence imaging-guided lymphadenectomy could increase the number of retrieved LNs, reduce intraoperative blood loss and the overall recurrence rate without increasing operative time and overall complications. It is very valuable for complete LNs dissection in radical gastrectomy for GC. Nevertheless, more high-quality prospective studies and RCTs are necessary to confirm this conclusion.

## Data availability statement

The original contributions presented in the study are included in the article/supplementary material. Further inquiries can be directed to the corresponding author.

## Author contributions

ZL made substantial contributions to conception and design for this work. BD, AZ, and ZL collected all the data. BD and ZL were the major contributors in writing the manuscript. YZ, WY, and LL performed critical revision for important intellectual content. All authors read and approved the final manuscript.

## Funding

This work was supported by Scientific Research Project of Southwest Medical University (No.2020ZRQNB026).

## Conflict of interest

The authors declare that the research was conducted in the absence of any commercial or financial relationships that could be construed as a potential conflict of interest.

## Publisher’s note

All claims expressed in this article are solely those of the authors and do not necessarily represent those of their affiliated organizations, or those of the publisher, the editors and the reviewers. Any product that may be evaluated in this article, or claim that may be made by its manufacturer, is not guaranteed or endorsed by the publisher.
